# Antimicrobial Activity of Nitric Oxide-Releasing Ti-6Al-4V Metal Oxide

**DOI:** 10.3390/jfb8020020

**Published:** 2017-06-21

**Authors:** Nina A. Reger, Wilson S. Meng, Ellen S. Gawalt

**Affiliations:** 1 Department of Chemistry and Biochemistry, Duquesne University, Pittsburgh, PA 15282, USA; regern@duq.edu; 2 Division of Pharmaceutical Sciences, Duquesne University, Pittsburgh, PA 15282, USA; meng@duq.edu; 3 McGowan Institute for Regenerative Medicine, University of Pittsburgh, Pittsburgh, PA 15282, USA

**Keywords:** Ti-6Al-4V, nitric oxide, self-assembled monolayers, *S*-nitroso-penicillamine, *Escherichia coli*, *Staphylococcus epidermidis*

## Abstract

Titanium and titanium alloy materials are commonly used in joint replacements, due to the high strength of the materials. Pathogenic microorganisms can easily adhere to the surface of the metal implant, leading to an increased potential for implant failure. The surface of a titanium-aluminum-vanadium (Ti-6Al-4V) metal oxide implant material was functionalized to deliver an small antibacterial molecule, nitric oxide. *S*-nitroso-penicillamine, a *S*-nitrosothiol nitric oxide donor, was covalently immobilized on the metal oxide surface using self-assembled monolayers. Infrared spectroscopy was used to confirm the attachment of the *S*-nitrosothiol donor to the Ti-Al-4V surface. Attachment of *S*-nitroso-penicillamine resulted in a nitric oxide (NO) release of 89.6 ± 4.8 nmol/cm^2^ under physiological conditions. This low concentration of nitric oxide reduced *Escherichia coli* and *Staphylococcus epidermidis* growth by 41.5 ± 1.2% and 25.3 ± 0.6%, respectively. Combining the *S*-nitrosothiol releasing Ti-6Al-4V with tetracycline, a commonly-prescribed antibiotic, increased the effectiveness of the antibiotic by 35.4 ± 1.3%, which allows for lower doses of antibiotics to be used. A synergistic effect of ampicillin with *S*-nitroso-penicillamine-modified Ti-6Al-4V against *S. epidermidis* was not observed. The functionalized Ti-6Al-4V surface was not cytotoxic to mouse fibroblasts.

## 1. Introduction

Titanium and its alloys have been utilized as a joint replacement material for dental, cardiovascular, and structural applications, due to strength of the materials, biocompatibility, and corrosion resistance. Titanium-aluminum-vanadium (Ti-6Al-4V) alloy was developed as an alternative to pure titanium as it has an increased elastic modulus, yield strength, and ultimate strength, factors important for the development of a metallic implant material [1,2]. Ti-6Al-4V implant materials are utilized in hip and knee joints, where hard tissue has failed [3]. In these applications, tissue regeneration and osseointegration is important to reduce implant wear, rejection, and failure [2,4]. However, implant rejection and failure can also result from infection [3,5,6]. If bacterial adhesion occurs before the tissue regeneration process after implantation, the host defense cannot prevent the surface colonization of bacteria, potentially leading to biofilm formation and infection [3]. Systemic antibiotics are often delivered in the hours leading up to a surgical procedure, but 90% of all implants show pathogenic microorganism adhesion post-implantation [5,7]. Thus, inhibiting initial bacterial adhesion through anti-infective implant materials is essential to preventing implant failure and rejection [3].

The continuous and systemic antibiotic therapies have increased the prevalence of antibiotic resistance, as over 2 million individuals in the United States annually contract an antibiotic-resistant infection [8]. A promising antimicrobial alternative, nitric oxide (NO), has been utilized in many site-specific delivery applications, through the direct modification of nanoparticles and various biomaterials [9,10,11,12,13,14,15,16]. The small, non-polar nature of NO allows it to easily cross the bacterial cell membrane, even in cases of antibiotic-resistant cells where the cell membrane has thickened (i.e., methicillin-resistant *Staphylococcus aureus* (MRSA)) [17]. The antibacterial effect of NO is due to oxidative and nitrosative stress it causes on the DNA, cell membrane, and proteins of a bacterial cell [18,19]. Low concentrations of NO have also been shown to trigger biofilm dispersal events and increase the effectiveness of a low concentration of antibiotic [18,20].

Unfortunately, the short half-life of NO makes delivery and storage difficult [10,21]. Thus, NO is typically delivered through a donor, such as *N*-diazeniumdiolates (NONOates) and *S*-nitrosothiols. Most nanoparticles and biomaterials are functionalized with NONOates, as the release of NO from these donors is tailorable and predictable upon protonation. Unfortunately, NONOates have the potential to decompose into potentially carcinogenic nitrosamines following NO release [22]. *S*-Nitrosothiols are natural NO donors, as the immune system produces these donors in response to a bacterial infection. These donors are light- and temperature-sensitive and are, therefore, more difficult to work with, but offer no harmful side effects or chance of antimicrobial resistance. 

NONOate donors have previously been presented at the surface of metallic implants using physisorption and synthetic polymeric coating methods [11,12,23]. Direct modification of a metallic surface to release NO was reported by Gallo and Mani [23]. Cobalt-chromium stents were modified with phosphonoacetic acid, followed by diethylenetriame (DETA)/NONOate. Although these stents were not tested against bacterial cultures, the modified metal surface released micromolar (µM) amounts of NO in vitro [23]. Nablo and coworkers generated a stainless steel implant coated with a silica sol-gel containing a NONOate donor that released picomole amounts of NO over 24 h, while reducing *Staphylococcal* bacterial adhesion onto the coated surface [12]. Holt et al. coated the surface of commercially-pure titanium fixation pins utilizing a silica xerogel containing a NONOate functionality. Like Nablo and coworkers, these titanium pins released similar amounts of NO and reduced bacterial adhesion 48 days post implantation in a mouse model [11,12]. Although these polymeric coatings seem promising, the long-term cytotoxicity of these NONOate silica gel coatings were not discussed, and could impose limitations on in vivo applications [11,12]. 

Antimicrobial compounds, such as antibiotics [24,25,26,27] and antimicrobial peptides [28,29], have been presented at the surface of metallic and ceramic biomaterials using self-assembled monolayers (SAMs) to reduce bacterial adhesion and colonization. SAMs are ordered molecular assemblies that form spontaneously via chemisorption onto an oxide-rich material and are composed of a head group, alkyl chain, and a tail group [30,31]. The head group strongly binds to the metal implant surface, the alkyl chain provides stability, and the tail group can be functionalized with bioactive molecules at the interface [30,31]. Thus, SAMs can be utilized to immobilize NO donors to the surface of metallic implant materials. This approach presents bioactive molecules on the surface while preserving the physical properties of the implanted metal material [32]. These SAMs strongly adhere to the metal oxide surface and remain through solvent and water rinsing and sonication and physical tests, including scratch and tape tests [33]. 

**Scheme 1:**
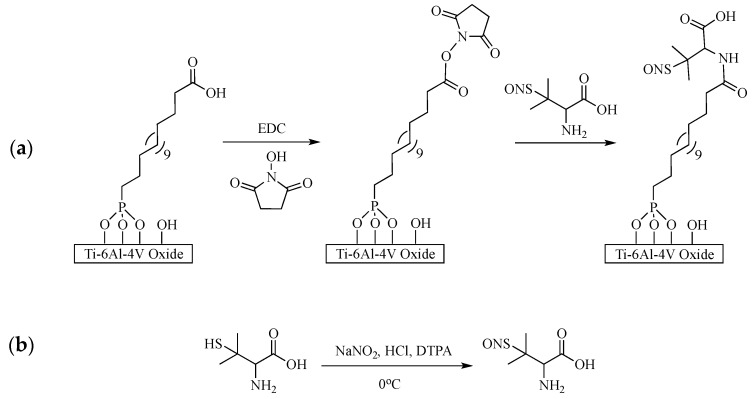
(**a**) The two-step reaction sequence for the immobilization of *S*-nitroso-penicillamine to the surface of COOH-Pa modified Ti-6Al-4V. The carboxylic acid group is first activated with ethylenediaminecarbodiimide (EDC)/N-hydroxysuccinimide (NHS) and then reacted with *S*-nitroso-penicillamine to form an amide bond. (**b**) The formation of *S*-nitroso-penicillamine from the reaction of acidified nitrite with penicillamine in the presence of a metal chelator.

Here, a Ti-6Al-4V metal oxide implant material was modified at the surface to release nanomolar amounts of NO from a strongly-bound SAM. A reaction at the monolayer terminus provided an interface for further surface reactions in which an *S*-nitrosothiol NO donor was immobilized onto the surface (Scheme 1). Previous immobilization of NO donors to metallic surfaces has been completed through the use of silica coatings and physisorption. This study immobilizes the *S*-nitrosothiol donor at the interface of the metal surface and eliminates the use of silica coatings. Additionally, the use of *S*-nitrosothiols avoids the potentially harmful side effects of NONOate donors. The functionalized Ti-6Al-4V substrates were tested for antimicrobial properties and cytotoxic effects and have the potential to deliver an antimicrobial alternative directly to an infection site, limiting the chance of antibiotic resistance.

## 2. Materials and Methods

### 2.1. Materials

Titanium-aluminum-vanadium (Ti-6Al-4V composed of 90% titanium, 6% aluminum, and 4% vanadium) foil of a 0.52 mm thickness was purchased from Goodfellow, Inc. (Corapolis, PA, USA). 16-Phosphonohexadecanoic acid (COOH-Pa, 97%), *N*-hydroxysuccinimide (NHS), 1-(3-dimethylaminopropyl)-3-ethylcarbodiimide hydrochloride (EDC), d-penicillamine (98–101%), sodium nitrite (NaNO_2_, 99.999% trace metal), Dulbecco’s phosphate-buffered saline (pH = 7.4), and ampicillin sodium salt were purchased from Sigma-Aldrich (Saint Louis, MO, USA) and used as received. Diethylenetriamine pentaacetic acid (DTPA, >99.0%) was purchased from Fluka (Mexico City, Mexico). Hydrochloric acid (HCl), 2-propanol (Optima), and tetracycline hydrochloride were obtained from Fisher Scientific (Waltham, MA, USA), and used as received. Tetrahydrofuran (THF, Optima) was obtained from Fisher Scientific and was distilled over sodium and benzophenone before use. Ethanol (200 proof) and deionized water (ddH_2_O) was obtained from Duquesne University. The nitrate/nitrite colorimetric assay kit was purchased from Cayman Chemical Company (Ann Arbor, MI, USA). Lennox LB Media was obtained from MP Biomedicals, Inc. (Solon, OH, USA) and two capsules were dissolved per 50 mL of ddH_2_O and autoclaved at 121 °C before use. *Escherichia coli* (ATCC^®^ 25922), *Staphylococcus epidermidis* (ATCC^®^ 14990), and 3T3 Swiss Albino mouse embryo fibroblasts (CCL-92) were obtained from American Type Culture Collection (ATCC) (Manassas, VA, USA). Dulbecco’s Modified Eagle’s Medium (DMEM), fetal bovine serum (FBS), penicillin/streptavidin, and Trypsin/EDTA were purchased from LONZA (Protsmouth, NH, USA). The Live/Dead^®^ Cytotoxicity/Viability Assay Kit for mammalian cells was purchased from Life Technologies/Fisher Scientific (Waltham, MA, USA).

### 2.2. Methods

#### 2.2.1. Substrate Preparation and Monolayer Formation

Ti-6Al-4V foils were sanded using four decreasing grain size sand papers (150, 320, 400, and 600 grit) and then cut into 1 cm × 1 cm square coupons. The coupons were cleaned by sonication in acetone for 15-min, followed by immersion in boiling methanol for 15 min. The coupons were placed in a 60 °C oven overnight to dry before further use. 

SAMs of COOH-Pa were formed on the surface of cleaned Ti-6Al-4V using an aerosol spray deposition method. A 1 mM solution of COOH-Pa was prepared in distilled THF and was poured into a thin layer chromatography (TLC) sprayer (Sigma-Aldrich, Saint Louis, MO, USA). The TLC sprayer was used in conjunction with a stream of nitrogen gas to spray the 1 mM COOH-Pa solution onto the Ti-6Al-4V coupons in four different cycles. Each coupon was sprayed once with COOH-Pa, placed in the 60 °C oven for 30 min, and then sprayed again with COOH-Pa for a total of four cycles. The coupons were placed in the oven at 60 °C overnight to remove excess solvent before film characterization. The coupons were rinsed and sonicated with THF to test the mechanical stability of the formed SAM. 

#### 2.2.2. Immobilization of Nitric Oxide Donor

*S*-nitroso-penicillamine, the NO donor, was immobilized onto the Ti-6Al-4V metal oxide surface using a two-step approach (Scheme 1a). Coupons with stable COOH-Pa monolayers were immersed in a solution containing 20 mM EDC and 50 mM NHS in 2-propanol [24,34]. The reaction proceeded with nitrogen bubbling through the solution for 1.5 h, protected from light. The coupons were removed from solution and dried under vacuum (0.1 Torr) for 45 min before further reaction. 

*S*-nitroso-penicillamine was generated by reacting d-penicillamine with acidified nitrite. Briefly, 100 mM of penicillamine was reacted with 100 mM NaNO_2_ and 250 mM HCl in the presence of 100 μM DTPA (a metal chelator) in an ice bath for 1 h and 15 min, while protected from light (Scheme 1b) [35,36]. After RSNO formation, the *S*-nitroso-penicillamine solution was diluted with 200 proof ethanol. The activated COOH-Pa coupons were immersed in the ethanol solution for 1.5 h, with nitrogen bubbling through. The coupons were removed from the solution without a solvent meniscus and placed under vacuum (0.1 Torr) overnight to dry. 

#### 2.2.3. Characterization Techniques

##### Ultraviolet-Visible Spectrometric Analysis

Liquid *S*-nitroso-penicillamine was scanned from 800 nm to 250 nm using a Varian ultraviolet-visible (UV-VIS) spectrophotometer (Agilent Technologies, Santa Clara, CA, USA) to determine the wavelength of maximum absorbance and the purity of the formed *S*-nitrosothiol compound. *S*-nitroso-penicillamine was diluted 1:1 with ddH_2_O to reduce signal saturation. 

##### Infrared Spectroscopy

SAM formation and stability, as well as the activation and immobilization steps of *S*-nitroso-penicillamine on Ti-6Al-4V, was characterized using a Nexus 470 Fourier Transform Infrared (FTIR) spectrometer (Nicolet Instruments, Madison, Wisconsin, USA) with a diffuse reflectance infrared Fourier transform (DRIFT) attachment. Each sample was analyzed under inert conditions with 256 scans (4000–400 cm^−1^) and a 4 cm^−1^ resolution. Clean, unmodified Ti-6Al-4V was used as a background before sample collection. 

##### In Vitro Nitric Oxide Release

NO release from *S*-nitroso-penicillamine-modified Ti-6Al-4V was monitored over 48 h using the Griess assay [23,37]. Briefly, three coupons of *S*-nitroso-penicillamine-modified Ti-6Al-4V were added to three separate vials containing 900 μL of PBS and placed on an incubator/shaker at 37 °C, while protected from light. The addition of PBS begins the decomposition process of *S*-nitrosothiols, as it contains low concentrations of metals that trigger NO release [38]. At each time point, the PBS was collected and frozen at −20 °C until assay preparation. The same volume of PBS that was removed at each time point was replaced [23]. 

The nitrate/nitrite colorimetric assay included reagents that allowed for the construction of a nitrate/nitrite calibration curve. Briefly, 80 μL of the thawed NO/PBS sample was incubated with 10 μL of enzyme cofactor mixture and 10 μL of nitrate reductase mixture. After incubation for 1 h, 50 μL of Griess Reagent R1 and 50 μL of Griess Reagent R2 were added to each well. The well plate was incubated at room temperature for 10 min before being read using an Infinite M1000 microplate reader (Tecan, Morrisville, NC, USA) at an absorbance of 540 nm. The concentration of NO was determined by the calibration curve and equation provided by Cayman Chemical (*n* = 9) [39]. 

#### 2.2.4. Bacterial Turbidity

Bacterial optical density was used to monitor the effectiveness of NO release from Ti-6Al-4V coupons against planktonic cultures of *E. coli* and *S. epidermidis* (ATCC, Manassas, VA, USA) in Lysogeny broth (LB) media. An overnight culture of *E. coli* or *S. epidermidis* was diluted to an OD_600nm_ of 0.1. In each well of a 24-well plate, 100 μL PBS, and 900 μL of planktonic OD_600nm_ *E. coli* or *S. epidermidis* culture was added. Three wells each contained: (i) *E. coli*/*S. epidermidis*, (ii) *E. coli*/*S. epidermidis* + unmodified Ti-6Al-4V, or (iii) *E. coli*/*S. epidermidis* + *S*-nitroso-penicillamine-modified Ti-6Al-4V. The plate was placed in a shaker incubator (230 rpm for *S. epidermidis* reduces bacterial adhesion to the surface of Ti-6Al-4V) at 37 °C for 2.5 h while protected from light. After incubation, the OD_600nm_ was collected for each well using a Varian UV-VIS spectrophotometer with three replicates per well (*n* = 9). LB media containing PBS was utilized as the blank.

#### 2.2.5. Antibiotic Susceptibility

The effectiveness of *S*-nitroso-penicillamine-modified Ti-6Al-4V in conjunction with commonly-prescribed antibiotics, tetracycline and ampicillin, were tested [18,40]. The concentration of tetracycline used was 100 ng/mL and used in experiments involving *E. coli*. Ampicillin at a concentration of 400 ng/mL was utilized in experiments involving *S. epidermidis*. Both antibiotic concentrations were previously determined in the laboratory and were chosen because of the ability to slightly inhibit bacterial growth, not completely inhibit it [18,20]. An overnight culture of *E. coli* or *S. epidermidis* was diluted to a 0.1 OD_600nm_. To each of three wells, 900 μL of diluted planktonic *E. coli* or *S. epidermidis* culture was placed in each well of a 24-well plate, with: (i) *E. coli*/*S. epidermidis* + 100 μL PBS, (ii) *E. coli*/*S. epidermidis* + 100 μL tetracycline/ampicillin + unmodified Ti-6Al-4V or (iii) *E. coli*/*S. epidermidis* + 100 μL tetracycline/ampicillin + *S*-nitroso-penicillamine-modified Ti-6Al-4V. The plate was incubated for 2.5 h at 37 °C under shaking conditions (230 rpm for *S. epidermidis* cultures). The bacterial turbidity of each well was collected using the Varian UV-Vis spectrophotometer at 600 nm, with three replicates of each well and LB media with the antibiotic/PBS subtracted as the blank (*n* = 9). 

#### 2.2.6. In Vitro Cytotoxicity of NO-Releasing Ti-6Al-4V

The cytotoxicity of *S*-nitroso-penicillamine-modified Ti-6Al-4V was determined against NIH3T3 Swiss Albino mouse embryo fibroblasts. The cells were grown in DMEM supplemented with 10% FBS and 1% penicillin/streptavidin. The fibroblasts were cultured until approximately 80% confluent. The cells were trypsinized from the flask, counted, and diluted to a concentration of 10,000 cells per mL of fresh DMEM. Three of each type of coupon, either unmodified or *S*-nitroso-penicillamine-modified Ti-6Al-4V, was placed in a well of a 24 well plate and 1 mL of cell suspension was added. The plate was incubated for 24 h at 37 °C in a 5% carbon dioxide environment. 

After a 24 h incubation, the number of live and dead fibroblast cells on the Ti-6Al-4V surfaces was determined using a Live/Dead^®^ Viability/Cytotoxicity Kit. Five spots on each microscope slide with an area of 0.6 mm^2^ were imaged under 10× magnification using an Axioskop2 fluorescent microscope. The number of live and dead cells was counted on 45 images for each sample and control (*n* = 45). The percent viability for each Ti-6Al-4V sample was calculated by dividing the number of live cells by the total number of cells (live and dead) and was normalized to the fibroblast control [41].

#### 2.2.7. Statistics

Statistical analysis was performed using Origin 8.0 software. Where applicable, the Analysis of Variance (ANOVA) with a Bonferroni post-hoc test was used to determine the averages, standard errors, and statistical significance of the collected data at the *p* < 0.05 level of significance. All analyzed data is presented as the mean ± standard error and all experiments were repeated at least twice.

## 3. Results

### 3.1. Characterization of S-Nitroso-Penicillamine-Modified Ti-6Al-4V

#### 3.1.1. Self-Assembled Monolayer Formation

**Figure 1:**
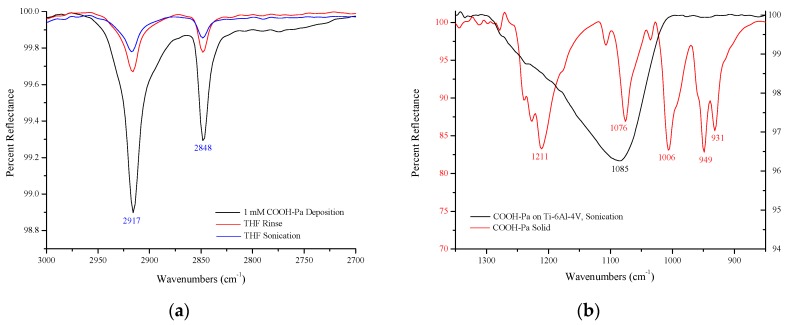

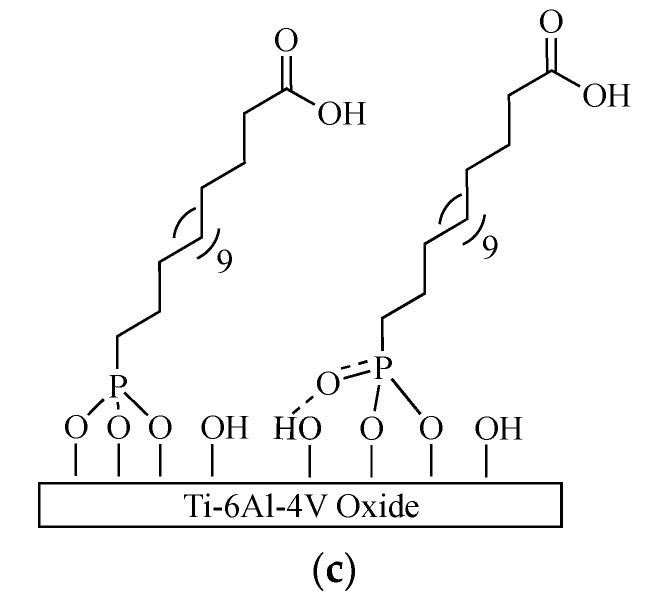
(**a**) Diffuse Reflectance Infrared Spectroscopy (DRIFT) spectra of the methylene stretching region of COOH-Pa on the surface of Titanium-Aluminum-Vanadium (Ti-6Al-4V) indicates an ordered, stable self-assembled monolayer (SAM) through sonication in solvent. (**b**) DRIFT spectra of the phosphonic acid head binding region, where the broad peak at 1085 cm^−1^ with a slight shoulder in the spectrum of the binding region of COOH-Pa confirms a mixture of bidentate and tridentate binding. (**c**) Illustration of the binding of the SAM to the oxide surface.

Methylene stretching peaks for an ordered monolayer are ν_CH2 asymmetric_ ≤ 2918 cm^−1^ and ν_CH2 symmetic_ ≤ 2850 cm^−1^ [31,33,42,43]. The methylene stretching region of the DRIFT spectrum of COOH-Pa on Ti-6Al-4V contains two peaks corresponding to the asymmetric and symmetric CH_2_ stretches, at 2917 and 2848 cm^−1^, respectively (Figure 1a). These peaks are indicative of a well-ordered film on the metal surface, characterized by alkyl chains in an all *trans*, crystalline-like conformation [20,31,33]. Additionally, the methylene stretches remain after solvent rinse and sonication, indicating a stable monolayer of COOH-Pa on the surface of Ti-6Al-4V [31]. 

The binding mode of COOH-Pa to the native oxide surface of Ti-6Al-4V was also examined using DRIFT (Figure 1b). When using organic acids containing termini of both carboxylic and phosphonic acid functional groups, the SAM preferentially forms with phosphonic acid as the head group and carboxylic acid group free at the tail [24,31,44]. The binding mode is determined by comparing solid COOH-Pa to that of the COOH-Pa monolayer on Ti-6Al-4V [24]. The spectrum of solid COOH-Pa contains peaks at 1214, 1077, 1008, 951, and 934 cm^−1^ corresponding to v_P=O_, v_P-O asymmetric_, v_P-O symmetric_, v_P-OH_, and v_P-OH_ of the phosphonic acid head group, respectively. The DRIFT spectrum of COOH-Pa bound to the Ti-6Al-4V surface contains one broad peak at 1085 cm^−1^ for v_P-O_. The loss of both P-OH peaks and the P=O peak, in combination with one broad P-O stretch, suggests a tridentate binding mode. However, a slight shoulder on the broad v_P-O_ peak could suggests some hydrogen bonding between the oxygen atom of the P=O stretch and the hydrogen of a surface hydroxyls, indicating an overall mixture of bidentate and tridentate binding is likely occurring (Figure 1c) [24].

#### 3.1.2. Immobilization of *S*-Nitroso-Penicillamine

**Figure 2:**
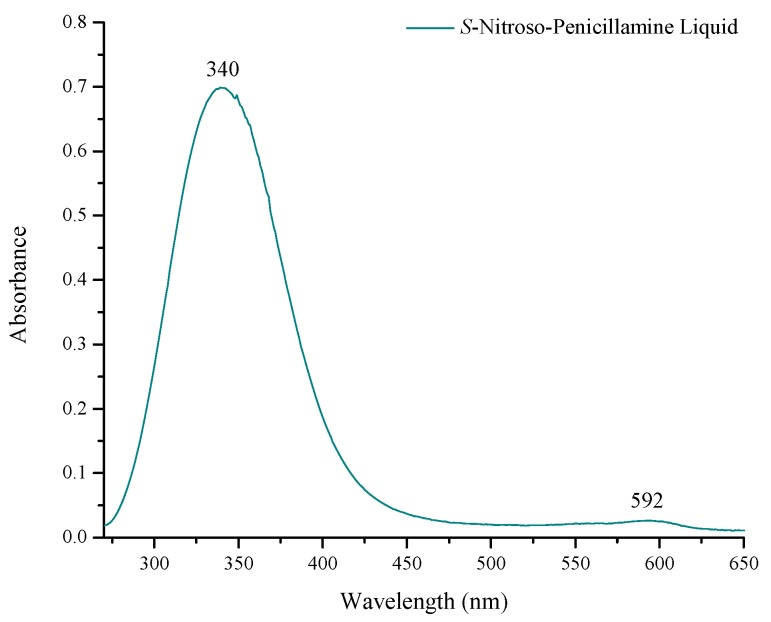
UV-VIS spectrum of the unbound *S*-nitroso-penicillamine liquid NO donor. The wavelength of the maximum absorbance at 342 nm and secondary absorbance at 595 nm confirms successful *S*-nitrosothiol formation.

UV-VIS spectroscopy was used to confirm the successful synthesis of *S*-nitroso-penicillamine, prior to surface immobilization. Absorbance maxima at 340 nm and 595 nm are indicative of the electronic transitions of π → π* and n_N_ → π*, respectively, for the SNO functionality in pure, unbound *S*-nitroso-penicillamine (Figure 2) [45,46,47]. Confirmation of this pure synthesis allows for further reaction, while the lack of the nitrite and nitrous acid signatures from the NO donor indicates that no excess nitrite or nitrous acid was present in the samples. The lack of interfering species is critical for the success of the NO release assay and the system itself.

**Figure 3:**
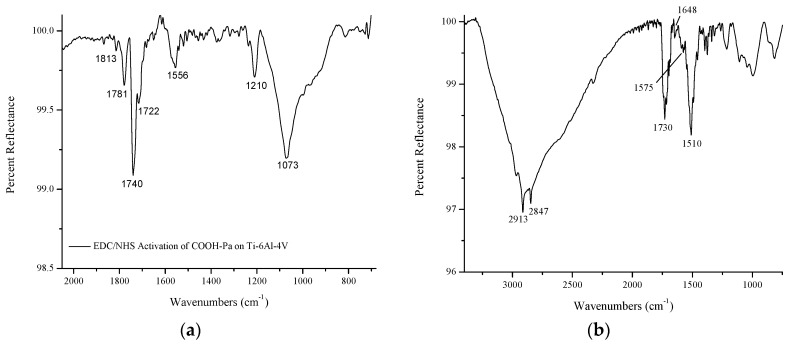
(**a**) DRIFT spectrum of EDC and NHS activation of carboxylic acid groups presented at the surface of the COOH-Pa monolayer on Ti-6Al-4V. Stretches for ester formation at 1813 cm^−1^, 1781 cm^−1^, and 1740 cm^−1^ indicated successful activation of the tail group for immobilization by amide bond formation. (**b**) DRIFT spectrum of the attachment of *S*-nitroso-penicillamine to the COOH-Pa modified Ti-6Al-4V surface as confirmed by the presence of amide I (1648 cm^−1^) and amide II (1575 cm^−1^). The NO group is present at 1510 cm^−1^ and the stretch at 1730 cm^−1^ indicates free carboxylic acid from the *S*-nitroso-penicillamine donor.

The immobilization of *S*-nitroso-penicillamine to the carboxylic acid tail of COOH-Pa was completed using carbodiimide coupling. The formation of a succinimidyl ester at the carboxylic acid tail indicates successful leaving group formation. The following peaks confirm the attachment of NHS to the carboxylic acid tail of COOH-Pa: 1813 cm^−1^ for ν_C=O_ for the NHS ester, 1781 cm^−1^ for ν_C=O_ for the symmetric stretch of the succinimidyl ester within the ring, and 1740 cm^−1^ ν_C=O_ for the asymmetric stretch of the succinimidyl ester within the ring (Figure 3a) [48]. The stretch at 1210 cm^−1^ is that of ν_C-O_ of the ester group [48]. Incomplete activation of the COOH-Pa carboxylic acid tails at the surface can be observed by the peak at 1722 cm^−1^ for ν_C=O_ [48]. Additionally, the stretch at 1556 cm^−1^ is that of an unreactive side product, *N*-acylurea [48]. The formation of this product is difficult to prevent under ambient conditions [48]. After this activation step, the phosphonic acid head group remains bound the surface in a primarily tridentate manner, based on the P-O stretch at 1075 cm^−1^.

The attachment of *S*-nitroso-penicillamine to the surface was completed through the formation of an amide bond. *S*-nitroso-penicillamine immobilization was confirmed by the presence of the ν_C=O_ amide I stretch at 1648 cm^−1^ and the ν_N-H_ amide II stretch at 1575 cm^−1^ (Figure 3b). The peak at 1730 cm^−1^ is that of carboxylic acid present in the *S*-nitroso-penicillamine NO donor, while the stretch at 1510 cm^−1^ for ν_N=O_ indicates that there is NO on the surface of Ti-6Al-4V. The methylene stretching peaks at 2913 cm^−1^ and 2847 cm^−1^ indicate that the COOH-Pa SAM is still adhered to the Ti-6Al-4V metal oxide surface.

### 3.2. Quantitation of NO Release from S-Nitroso-Penicillamine-Modified Ti-6Al-4V

**Figure 4:**
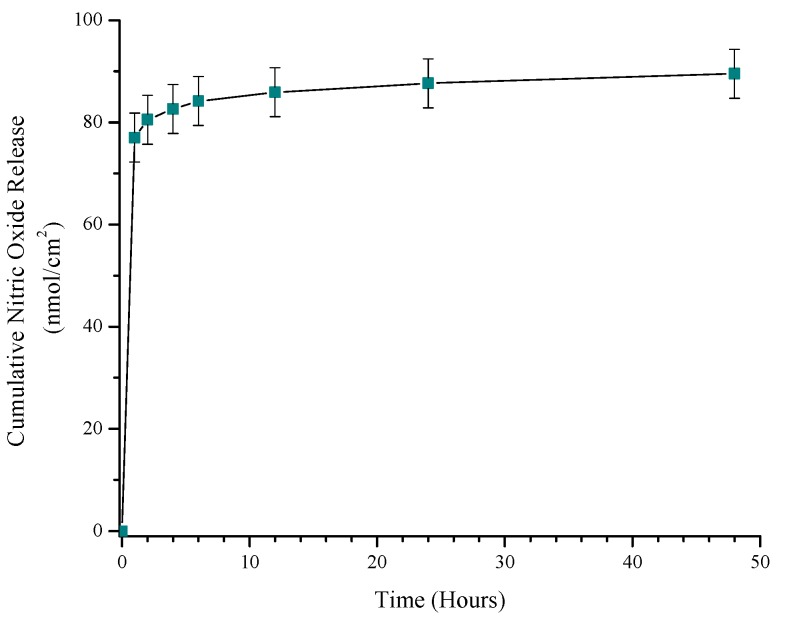
Quantitation of NO release from *S*-nitroso-penicillamine-modified Ti-6Al-4V, determined using a nitrate/nitrite colorimetric assay. The modified Ti-6Al-4V coupons released 89.6 ± 4.8 nmol nitric oxide (NO)/cm^2^ over a 48-h time period. Each data point expresses the average NO release ± standard error (SE).

The total amount of NO released from the surface of the modified Ti-6Al-4V was determined using the Griess assay, a nitrate/nitrite colorimetric assay. NO degrades rapidly into nitrate under physiological conditions; this assay converts nitrates to nitrites, and the total amount of nitrite was quantified to reflect the amount of NO released [23]. The total amount of NO released by *S*-nitroso-penicillamine-modified Ti-6Al-4V cumulatively over 48 h was 89.6 ± 4.8 nmol/cm^2^ (Figure 4). The NO was burst-released between the first and second hour of the assay, followed by a slow release of NO for the remainder of the assay.

### 3.3. Antimicrobial Effect of S-Nitroso-Penicillamine-Modified Ti-6Al-4V

#### 3.3.1. Bacterial Turbidity: *E. coli* and *S. epidermidis*

**Figure 5:**
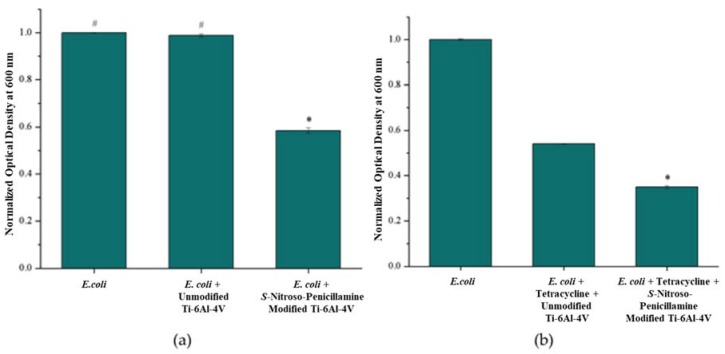
(**a**) Bacterial turbidity assay of *E. coli* cultures treated with *S*-nitroso-penicillamine-modified Ti-6Al-4V shown as the mean ± standard error, *p* < 0.05. *Escherichia coli (E. Coli)* and *E. coli* grown with unmodified Ti-6Al-4V substrates were not statistically different (#). *E. coli* inhibition by *S*-nitroso-penicillamine-modified Ti-6Al-4V is statistically different (*) than all other groups. (**b**) Bacterial turbidity assay of *Stapyloccocus epidermidis (S. epidermidis)* cultures treated with *S*-nitroso-penicillamine-modified Ti-6Al-4V. *S. epidermidis* and *S. epidermidis* grown with unmodified Ti-6Al-4V substrates were not statistically different (#), while *S. epidermidis* grown with *S*-nitroso-penicillamine-modified Ti-6Al-4V was statistically different (*) than all other groups.

The antibacterial activity of *S*-nitroso-penicillamine-modified Ti-6Al-4V against Gram-negative bacteria was assessed by an *E. coli* challenge using bacterial turbidity. The planktonic growth of *E. coli* incubated with Ti-6Al-4V coupons was monitored via optical density at 600 nm (OD_600nm_). After incubating a 0.1 OD_600nm_ *E. coli* culture with Ti-6Al-4V coupons for 2.5 h, the OD_600nm_ was collected for all *E. coli* controls and samples. The normalized average OD_600nm_ was 1.00 ± 0.002 for the *E. coli* control, 0.989 ± 0.007 for the unmodified Ti-6Al-4V control, and 0.585 ± 0.011 for *S*-nitroso-penicillamine-modified Ti-6Al-4V (Figure 5a). The OD_600nm_ of the *E. coli* incubated with *S*-nitroso-penicillamine-modified Ti-6Al-4V was statistically lower than both the *E. coli* control and *E. coli* grown with unmodified Ti-6Al-4V coupons. *E. coli* growth in the presence of *S*-nitroso-penicillamine modified substrates resulted in a 41.5 ± 1.2% growth reduction compared to the *E. coli* control. This confirms that *S*-nitroso-penicillamine is an active antimicrobial compound after being immobilized to the Ti-6Al-4V metal oxide surface. Additionally, *E. coli* grown with and without Ti-Al-4V coupons were statistically the same, indicating that *E. coli* cells are not adhering to the surface of the Ti-6Al-4V coupons.

The antibacterial activity of *S*-nitroso-penicillamine-modified Ti-6Al-4V surfaces against *S. epidermidis*, the Gram-positive bacteria, was assessed by a bacterial turbidity challenge. The OD_600nm_ was collected for all *S. epidermidis* controls and NO modified Ti-6Al-4V samples after a 2.5 h incubation. The average OD_600nm_ was 1.00 ± 0.005 for the *S. epidermidis* control, 0.985 ± 0.006 for the unmodified Ti-6Al-4V control, and 0.747 ± 0.005 for *S*-nitroso-penicillamine-modified Ti-6Al-4V (Figure 5b). The OD_600nm_ of the *S. epidermidis* incubated with *S*-nitroso-penicillamine-modified Ti-6Al-4V substrates was statistically lower than both the *S. epidermidis* control and *S. epidermidis* grown with unmodified Ti-6Al-4V, resulting in a 25.3 ± 0.6% growth reduction compared to the *S. epidermidis* control. The OD_600nm_ of *S. epidermidis* and *S. epidermidis* grown with unmodified Ti-6Al-4V were statistically the same, indicating that no bacterial cells were adhering to the Ti-6Al-4V surface.

#### 3.3.2. Antibiotic Susceptibility

**Figure 6:**
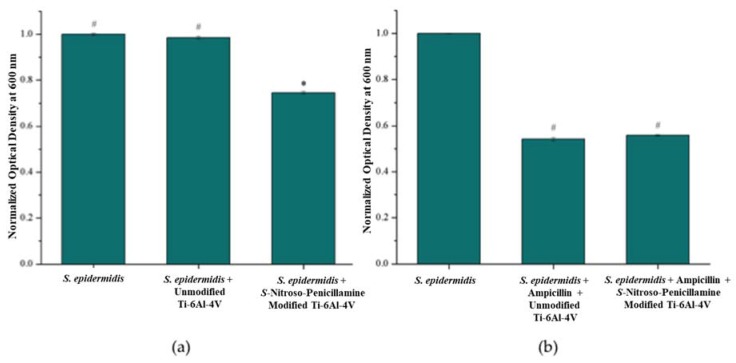
(**a**) Antibiotic susceptibility challenge in the presence of *E. coli* cultures treated with 100 ng/mL tetracycline and *S*-nitroso-penicillamine-modified Ti-6Al-4V, shown as mean ± standard error, *p* < 0.05. *E. coli* treated with *S*-nitroso-penicillamine-modified Ti-6Al-4V and tetracycline is statistically different (*) than all other groups. (**b**) Antibiotic susceptibility challenge of *S. epidermidis* cultures treated with 0.4 μg/mL ampicillin and *S*-nitroso-penicillamine-modified Ti-6Al-4V. *S. epidermidis* grown with *S*-nitroso-penicillamine-modified Ti-6Al-4V and ampicillin and *S. epidermidis* grown with unmodified Ti-6Al-4V and ampicillin were not statistically different (#).

The synergistic effect of the nanomolar amounts of NO released from the Ti-6Al-4V surface was tested in conjunction with 100 ng/mL tetracycline against *E. coli*. After 2.5 h, the normalized average OD_600nm_ for *E. coli* grown without tetracycline was 1.00 ± 0.002, *E. coli* grown with 100 ng/mL tetracycline and unmodified Ti-6Al-4V had an OD_600nm_ of 0.541 ± 0.001, and the average OD_600nm_ for *E. coli* cultures in the presence of *S*-nitroso-penicillamine-modified Ti-6Al-4V and tetracycline was 0.349 ± 0.007 (Figure 6a). The OD_600nm_ of *E. coli* incubated with *S*-nitroso-penicillamine-modified Ti-6Al-4V and tetracycline was statistically lower than the *E. coli* control and *E. coli* treated with tetracycline. The addition of *S*-nitroso-penicillamine Ti-6Al-4V increases the effectiveness of tetracycline by 35.4 ± 1.3% and, thus, a synergistic effect was observed.

The NO released from *S*-nitroso-penicillamine-modified Ti-6Al-4V increased the effectiveness of an antibiotic, tetracycline, against *E. coli*. The same effect was tested against *S. epidermidis* in the presence of ampicillin, an antibiotic effective against Gram-positive bacteria. After 2.5 h, the normalized average OD_600nm_ for *S. epidermidis* without ampicillin was 1.00 ± 5.0 × 10^−4^. *S. epidermidis* grown with 0.4 μg/mL ampicillin and unmodified Ti-6Al-4V had an OD_600nm_ of 0.542 ± 0.01 and the average OD_600nm_ for *S. epidermidis* grown with *S*-nitroso-penicillamine-modified Ti-6Al-4V and ampicillin was 0.559 ± 0.003 (Figure 6b). The OD_600nm_ of *S. epidermidis* incubated with *S*-nitroso-penicillamine-modified Ti-6Al-4V and ampicillin was not statistically different than *S. epidermidis* cultures grown with unmodified Ti-6Al-4V and ampicillin, indicating no synergistic effect against a Gram-positive bacterial species.

### 3.4. Cytotoxic Effect of S-Nitroso-Penicillamine-Modified Ti-6Al-4V

**Figure 7:**
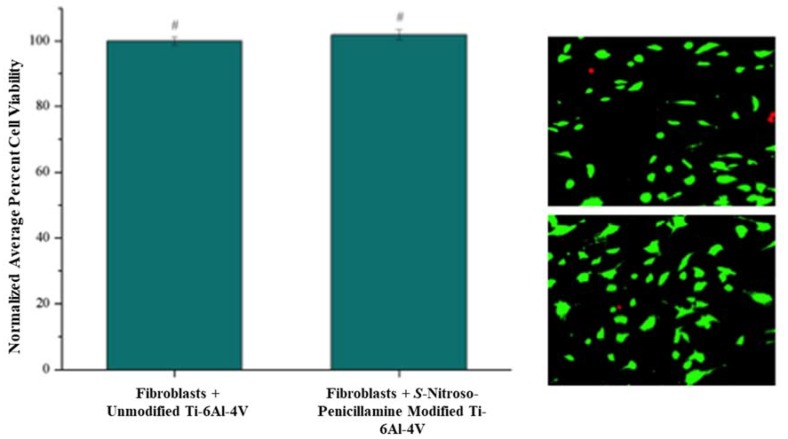
Normalized average percent cell viability of NIH3T3 mouse embryo fibroblasts grown on unmodified Ti-6Al-4V and *S*-nitroso-penicillamine-modified Ti-6Al-4V. Data is shown as the mean ± SE, *p* < 0.05. Fibroblast growth was not statistically different between Ti-6Al-4V and NO-releasing Ti-6Al-4V (#). The Live/Dead images of NIH3T3 fibroblasts are below, from top to bottom: fibroblasts grown on unmodified Ti-6Al-4V and fibroblasts grown on *S*-nitroso-penicillamine-modified Ti-6Al-4V.

The cell viability of NIH3T3 fibroblasts grown on *S*-nitroso-penicillamine-modified Ti-6Al-4V was determined using a Live/Dead^®^ Viability/Cytotoxicity assay. Live and dead cells were counted on each microscope image and the average percent viability was calculated [41]. The resulting percent viabilities were normalized to the fibroblasts grown on unmodified Ti-6Al-4V. The normalized average percent cell viability for fibroblasts grown on unmodified Ti-6Al-4V and *S*-nitroso-penicillamine-modified Ti-6Al-4V was 100.0 ± 1.3% (± Standard Error (SE)) and 101.9 ± 1.6%, respectively (Figure 7). Fibroblasts grown on *S*-nitroso-penicillamine-modified Ti-6Al-4V had statistically the same percent cell viability as the unmodified Ti-6Al-4V control, indicating that functionalization of the surface to release NO did not cause the Ti-6Al-4V substrates to be cytotoxic to mammalian cells.

## 4. Discussion

NO-releasing compounds have not been previously covalently immobilized onto the surface of metal oxide implant materials, but have been incorporated into polymeric matrices on the metal surface [11,12,23]. Most publications involving NO release from biomaterials, whether metallic, silicate, or polymeric, utilize potentially carcinogenic NONOate chemistry [9,10,11,12,21,23,49,50,51]. Here, the release of NO from a covalently-immobilized *S*-nitrosothiol donor from the surface of Ti-6Al-4V to reduce *E. coli* and *S. epidermidis* growth and increase the effectiveness of an antibiotic was investigated. This approach has the potential to be utilized in implant surgeries to reduce the colonization of bacteria post-implantation and ultimately, prevent implant rejection. 

The surface of Ti-6Al-4V was first modified with a COOH-Pa SAM. The phosphonic acid functional group was bound to the surface of Ti-6Al-4V using the thin oxide layer present at the metal surface. The methylene stretches of the alkyl chain in the DRIFT spectrum indicate that the SAM is ordered, giving optimal, crystalline packing of molecules on the surface (Figure 1a). This monolayer was also stable since it survived rinsing and sonication in solvent, which tests the mechanical and chemical stability. COOH-Pa was bound to the surface in a mixture of bidentate and tridentate binding, as seen by a broad stretch at 1085 cm^−1^ in the DRIFT spectrum with a shoulder indicating possible hydrogen bonding with the P=O of the head group with the oxide surface (Figure 1b) [24]. 

Since COOH-Pa was bound to the surface using the phosphonic head group, the carboxylic acid tail groups were available for carbodiimide coupling with the primary amine functionality within *S*-nitroso-penicillamine. *S*-nitroso-penicillamine contains both a primary amine and carboxylic acid functionality; therefore, completing the coupling reaction in a single mixture would have likely caused amide bond formation between individual *S*-nitroso-penicillamine molecules, and not to the surface carboxylic acid groups presented from COOH-Pa. As a result, the COOH-Pa modified Ti-6Al-4V required EDC/NHS activation before reacting with the NO donor. The tail of the COOH-Pa SAM was activated with EDC and NHS to present NHS at the surface as a more effective leaving group during amide bond formation. Activation was confirmed by the presence of stretches at 1813 cm^−1^ for ν_C=O_ for the NHS ester, 1781 cm^−1^ for ν_C=O_ for the symmetric stretch of the succinimidyl ester within the ring, and 1740 cm^−1^ ν_C=O_ for the asymmetric stretch of the succinimidyl ester within the ring in the DRIFT spectrum (Figure 3a). 

The synthesis of the *S*-nitrosothiol donor was confirmed using UV-VIS, where the wavelength of maximum absorbance at 340 nm matches that of the characteristic SNO group of other well characterized *S*-nitrosothiol donors (Figure 2) [46,47,52]. The UV-VIs spectrum of unbound *S*-nitroso-penicillamine lacks contributions from excess nitrite and nitrous acid from the synthesis step and, thus, could be utilized in further reactions and NO release assays [53]. 

Reaction of the EDC/NHS activated COOH-Pa modified Ti-6Al-4V coupons with *S*-nitroso-penicillamine showed two stretches in the infrared spectrum at 1648 cm^−1^ and 1575 cm^−1^ indicative of amide I and amide II bond formation, respectively, indicating successful *S*-nitroso-penicillamine attachment to the surface (Figure 3b). Additionally, the NO moiety at 1510 cm^−1^ in the infrared spectrum further confirm attachment to the metal oxide surface. The peak at 1730 cm^−1^ is a multiplet and is due to the unreacted carboxylic acid group of *S*-nitroso-penicillamine and unreacted carboxylic acid groups at the SAM termini. The *N*-acylurea present after the activation step does not appear in the infrared spectrum of *S*-nitroso-penicillamine attachment, indicating that the unreactive product was likely physisorbed onto the surface of the metal. The multiple small peaks within the 1730 cm^−1^ stretch could also indicate some unreacted carboxylic acid groups from the COOH-Pa presented at the metal surface, as observed during the activation step. 

The amount of NO released from the surface of *S*-nitroso-penicillamine-modified Ti-6Al-4V was determined through the Griess assay, which measures the amount of NO as it is converted from nitrate to nitrite. From this assay, it was determined that 89.6 ± 4.8 nmol of NO was released per cm^2^ (Figure 4). This amount of NO is less than the μM levels of NO reported by Gallo et al. [23], but more than the pmole/cm^2^ s amounts reported by Nablo et al. [12] and Holt et al. [11]. *S*-nitroso-penicillamine is a tertiary NO donor, which is the most stable of the *S*-nitrosothiol donors, and is likely responsible for the slow, increasing release of NO over the 48-time period. 

Unbound *S*-nitroso-penicillamine was found to exhibit inhibitory effects against both *E. coli* and *S. epidermidis*. When immobilized, *S*-nitroso-penicillamine maintained its antimicrobial activity like the original, unbound molecule. The nanomolar amount of NO released from *S*-nitroso-penicillamine-modified Ti-6Al-4V had a bacteriostatic effect against both *E. coli* and *S. epidermidis* cultures, as observed from the results of the bacterial turbidity assay. *E. coli* growth was inhibited by 41.5 ± 1.2% when grown in the presence of *S*-nitroso-penicillamine-modified Ti-6Al-4V, which is significantly different than the *E. coli* controls (Figure 5a). *S*-nitroso-penicillamine-modified Ti-6Al-4V inhibited *S. epidermidis* growth by 25.3 ± 0.6%, which is significantly different than the *S. epidermidis* controls. *S*-nitroso-penicillamine is not as bacteriostatic against *S. epidermidis* when compared to *E. coli*, but Gram-positive bacteria, in general, are more difficult to inhibit with NO (Figure 5b) [54].

*S*-nitroso-penicillamine-modified Ti-6Al-4V showed a synergistic effect against *E. coli* when coupled with 100 ng/mL tetracycline, resulting in a 35.4 ± 1.3% reduction in growth when compared to *E. coli* grown with tetracycline (Figure 6a). NO is thought to change the physiology of Gram-negative cells, making them more susceptible to antibiotic treatment [18]. The NO-releasing film on the metal oxide developed here is capable of increasing the effectiveness of an antibiotic and, thus, less antibiotic would be needed to treat an *E. coli* infection, subsequently reducing the risk of antibiotic resistance. 

Unfortunately, *S*-nitroso-penicillamine-modified Ti-6Al-4V did not exhibit a synergistic effect against *S. epidermidis* when coupled with ampicillin or other antibiotics (data not shown) (Figure 6b). Gram-positive bacteria produce their own nanomolar amounts of NO through bacterial nitric oxide synthase as a defense mechanism to oxidative stress, antimicrobial treatments, and other bacterial species [54]. NO production in a Gram-positive bacterial culture increases as the cells are treated with an antibiotic [54]. Thus, the exogenously-delivered nanomolar amounts of NO from the Ti-6Al-4V surface in the presence of ampicillin does not inhibit bacterial growth because the bacteria within the culture are already making their own NO [54].

To consider this *S*-nitrosthiol-modified Ti-6Al-4V as a metallic implant material, the metal surface should not be cytotoxic to mammalian cells in vitro. Though *S*-nitroso-penicillamine-modified Ti-6Al-4V inhibits bacterial cells, the modified Ti-6Al-4V should not inhibit mouse fibroblast growth. *S*-nitroso-penicillamine-modified Ti-6Al-4V showed no cytotoxic effect to fibroblasts after 24 h, as the percent viability of fibroblasts grown on *S*-nitroso-penicillamine-modified Ti-6Al-4V and unmodified Ti-6Al-4V were statistically the same (Figure 7).

## 5. Conclusions

A *S*-nitrosthiol donor, *S*-nitroso-penicillamine, was immobilized to the surface of a Ti-6Al-4V metal oxide implant material using SAMs and carbodiimide coupling. The thin oxide layer on the surface of Ti-6Al-4V was reacted with an organic acid to form a functional monolayer on the metal oxide surface. The organic acid presented a carboxylic acid functional group at the surface, allowing for amide bond formation between the primary amine of *S*-nitroso-penicillamine. The *S*-nitrosothiol modified Ti-6Al-4V released nanomolar amounts of NO in vitro and was bacteriostatic against *E. coli* and *S. epidermidis*, indicating that the antimicrobial activity of *S*-nitroso-penicillamine was maintained after immobilization to the Ti-6Al-4V surface. *S*-nitroso-penicillamine-modified Ti-6Al-4V increased the effectiveness of tetracycline against *E. coli*, potentially allowing for the use of lower concentrations of antibiotics. The same synergistic effect in conjunction with ampicillin against *S. epidermidis* was not observed, as *Staphylococci* species are capable of producing their own NO when treated with antibiotics.
